# Effects of three different types of anaesthesia on perioperative bleeding control in functional endoscopic sinus surgery

**DOI:** 10.1007/s00405-012-2311-1

**Published:** 2012-12-22

**Authors:** Jarosław Miłoński, Hanna Zielińska-Bliźniewska, Wojciech Golusiński, Joanna Urbaniak, Rafał Sobański, Jurek Olszewski

**Affiliations:** 1 Department of Otolaryngology and Laryngological Oncology, 2nd Chair of Otolaryngology, Medical University of Lodz, Zeromskiego 113, 90-549 Lodz, Poland; 2 Department of Head and Neck Surgery and Laryngological Oncology, Medical University of Poznan, Poznan, Poland; 3 Department of Anaesthesiology and Intensive Therapy, Medical University of Lodz, Lodz, Poland

**Keywords:** Type of anaesthesia, Perioperative bleeding, Functional endoscopic sinus surgery (FESS)

## Abstract

The aim of the study was to assess the effect of three different types of anaesthesia on perioperative bleeding control and to analyse the mean arterial blood pressure and heart rate in patients undergoing endoscopic paranasal sinus surgery. Ninety patients (30 women and 60 men, aged 18–85 years) scheduled to undergo functional endoscopic sinus surgery in the years 2008–2010 were identified as candidates for inclusion in the study. Patients were randomly assigned to one of three groups (30 patients each) according to the type of general anaesthesia to be administered. Groups I and II both received inhalation anaesthesia (sevoflurane for sedation) and intravenous anaesthesia (fentanyl in group I, remifentanil in group II). Anaesthesia was delivered solely via intravenous route (TIVA) in group III, with propofol used for sedation and remifentanil for analgesia. Blood pressure and heart rate were monitored during surgery and post-surgically for 4 h. Mean anaesthesia duration in groups I, II and III was 108.7 ± 20.8, 112.6 ± 22.2 and 103.7 ± 17.5 min and the surgery duration was 71.3 ± 16.7, 78.8 ± 24.2 and 66.5 ± 15.5 min, respectively. Mean blood loss during surgery was 365.0 ± 176.2, 340.0 ± 150.5 and 225.0 ± 91.7 ml, with a mean blood loss rate of 5.1 ± 2.4, 4.5 ± 2.2 and 3.4 ± 1.1 ml/min in groups I, II and III, respectively. Technologically advanced control of the drug dose with the TIVA technique allows for better control of perioperative bleeding.

## Introduction

Excessive bleeding during extensive endoscopic surgery of the paranasal sinuses can compromise the safety and efficiency of the surgical procedure. To ensure hemodynamic balance and patient safety, it is essential to monitor and control perioperative bleeding. Fortunately, the amount of blood lost during surgery can be easily determined by measuring the total quantity of blood suctioned from the operative field.

Good surgical field visibility is one of the basic prerequisites for a precise and safe otolaryngological operation, and the main obstacle to good visibility is excessive perioperative bleeding. Several factors, including arterial blood pressure, heart rate and coagulation disorders, have a large impact on perioperative bleeding. For this reason, every effort should be made to maintain these cardiovascular parameters at low levels.

One of the main methods of reducing perioperative bleeding during functional endoscopic sinus surgery (FESS) is the use of controlled hypotension. However, poorly controlled hypotension can lead to lower blood flow to organs that are sensitive to fluctuations in perfusion pressure. The use of precisely dosed modern anaesthetic agents, together with proper patient positioning, allows us to manage haemodynamic parameters during surgery [1] and thus to easily control hypotension. Although several such modern anaesthetic agents and methods are available, one method in particular—total intravenous anaesthesia (TIVA)—has become increasingly popular in recent years [2–6]. In our practice, we have been using TIVA with great success for several years now. However, few studies have evaluated the impact of TIVA on perioperative bleeding.

The aim of the present study was to compare TIVA to two other types of conventional anaesthesia delivery in order to assess the impact on perioperative bleeding control and on mean arterial pressure and heart rate in patients before, during and after endoscopic paranasal sinus surgery.

## Materials and methods

In the 2-year period from 2008 to 2010, 502 patients (209 women [41.6 %] and 293 men [58.4 %]), aged 18–85 years underwent FESS at the Department of Otolaryngology of the Military Medical Academy in Lodz. Of these 502 patients, 90 (30 women and 60 men) were randomly selected to participate in this randomized, prospective study. The women ranged in age from 18 to 75 years (mean 46.6 ± 11.12) and the men from 20 to 85 years (mean 54.4 ± 13.29). The inclusion criteria were based on the American Society of Anesthesiologists (ASA) Physical Status Scale Ratings [6] and were the same for all analgesic procedures.

The patients were allocated to one of three groups (30 patients each) based on the intended general anaesthesia approach (i.e. intubation, mechanical ventilation, or life and ventilation parameters monitoring). Both inhalation anaesthesia (sevoflurane for sedation) and intravenous anaesthesia were used for patients in groups I and II. The only difference between groups I and II was in the intravenous anaesthetic agent used (fentanyl in group I, remifentanil in group II). Sevoflurane concentration was regulated by an evaporator and monitored in expired gases. The third group (group III) received anaesthesia administered solely via the intravenous route (TIVA), with propofol used for sedation and remifentanil for analgesia.

Target-controlled infusion (TCI) was used to deliver the anaesthetics in group III, with the infusion pump programmed in the usual manner. Briefly, this is done by entering the value of the target plasma drug level along with the patient’s age and weight. The device contains the appropriate pharmacokinetic profile and, using these input data, calculates and adjusts (several times per minute) the correct speed for intravenous drug delivery to achieve the targeted plasma level. The pharmacokinetic profile programmed in the pump is specific for each company and development of this profile is based on several thousand studies of plasma drug concentration during anaesthesia in patients of various ages and weights [6, 7].

For intubation, the same muscle relaxant was used in all three groups. Controlled hypotension was applied to maintain the systolic blood pressure below 100 mm Hg, with analgesic and sedative doses administered accordingly.

The following parameters were assessed: duration of anaesthesia, duration of surgery, total perioperative blood loss and perioperative blood loss rate (ml/min). Patients who met the criteria for surgery were randomly allocated to one of the three groups. All patients were ASA class one and two for general anaesthesia risk. Surgery was performed in the reverse Trendelenburg position, at a 15°–20° angle, without use of a shaver.

Inclusion criteria for surgery were as follows: arterial blood pressure ≤140/90 mm Hg and general anaesthesia risk ≤class 2 on the ASA scale. Patients took antihypertensive agents up to the day of the surgery to ensure a stable blood pressure during the perioperative period [8].

On hospital admission, blood pressure was measured and a clinical examination was performed to assess cardiovascular status and the possible need for additional treatment. In addition, the following laboratory tests were performed: blood cell counts, coagulogram, ESR (erythrocyte sedimentation rate) and CRP (C-reactive protein). All patients underwent a CT scan of the paranasal sinuses and, depending on comorbidities and blood type, additional tests were performed as necessary.

All patients received premedication (benzodiazepine) to minimize the impact of the sympathetic nervous system on the cardiovascular system. All surgical interventions were conducted under thermally induced voltage alterations with remifentanil and propofol (anaesthesia induction). Arterial blood pressure and heart rate were monitored continuously during surgery. Patients remained under nursing care during the postoperative period. Cardiovascular system parameters were monitored every 15 min for at least 4 h following surgery. The mean arterial blood pressure (MAP) and heart rate (HR) values, as well as standard deviations, were calculated.

### Statistical analysis

Results were analysed with Chi-squared tests, with a significance level of *p* < 0.05. The Student’s *t* test was used for inter-group comparisons. Tukey’s HSD post hoc test was used to compare more than one group. Pearson’s correlation coefficient was used to assess the strength of linear relationship between the variables.

## Results

A total of 30 women and 60 men were included in the study. Group I consisted of 8 (26.7 %) women and 22 (36.7 %) men; group II 9 (30.0 %) women and 21 (35.0 %) men; and group III 13 (43.3 %) women and 17 (28.3 %) men. The statistical analysis showed no significant differences between men and women in total blood loss or blood loss rate. At baseline, 55 of the 90 patients (61.1 %) had normal arterial blood pressure, 25 (27.8 %) were receiving treatment for hypertension and 10 (11.1 %) had untreated hypertension.

**Fig. 1:**
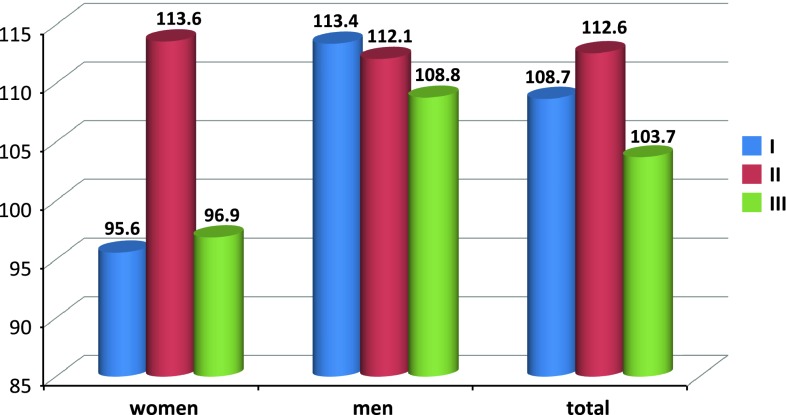
Mean anaesthesia duration (min) in individual anaesthesia types by patient gender

**Fig. 2:**
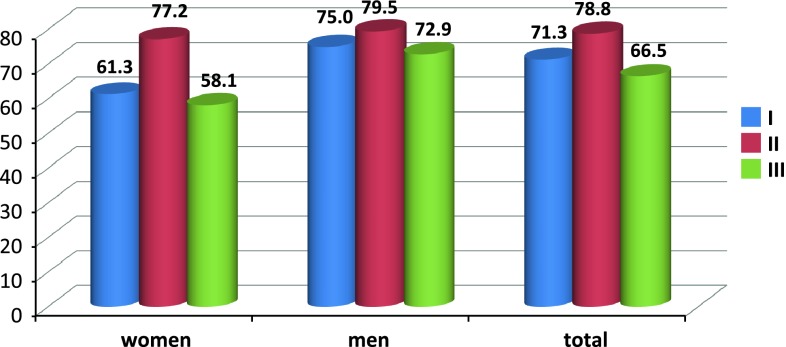
Mean surgery duration (min) in individual anaesthesia types by patient gender

The mean duration of anaesthesia was as follows (Fig. 1): group I, 108.7 ± 20.8 min; group II, 112.6 ± 22.2 min; and group III, 103.7 ± 17.5 min. No significant difference between the type of anaesthesia and duration thereof was observed. Surgical time was as follows (Fig. 2): group I, 71.3 ± 16.7 min; group II, 78.8 ± 24.2 min; and group III, 66.5 ± 15.5 min. Tukey’s HSD post hoc test revealed a statistically significant association between anaesthesia type and surgical duration: surgical time was significantly less in group III versus groups I and II (*p* < 0.05; coefficient of determination, *R* ^2^ = 6.7 %). No significant differences were observed in surgical time between groups I and II.

**Fig. 3:**
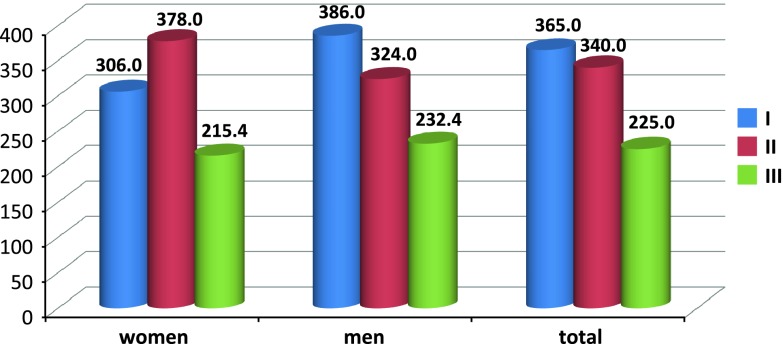
Mean values of blood loss (ml) in particular anaesthesia types by patient gender

**Fig. 4:**
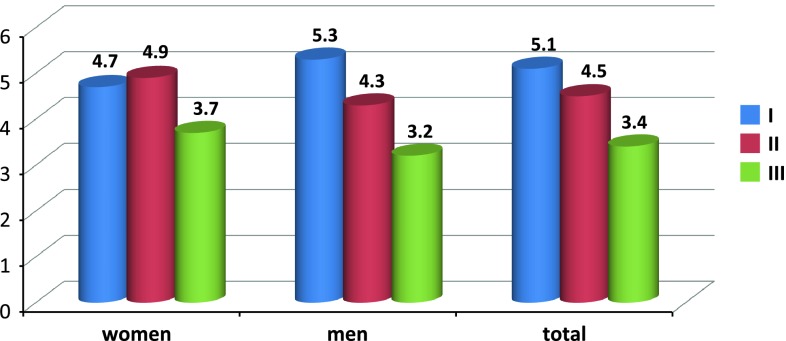
Rate of blood loss (ml/min) in individual anaesthesia types by patient gender

Mean blood loss during surgery was as follows (Fig. 3): group I, 365.0 ± 176.2 ml; group II, 340.0 ± 150.5 ml; and group III, 225.0 ± 91.7 ml. Mean blood loss was significantly lower in group III compared to groups I and II (*p* < 0.00; coefficient of determination, *R* ^2^ = 15.7 %); no significant differences between groups I and II were observed for this variable. Mean blood loss rates were as follows: group I, 5.1 ± 2.4 ml/min; group II, 4.5 ± 2.2 ml/min; and group III, 3.4 ± 1.1 ml/min (Fig. 4). The rate of blood loss/min was significantly lower in group III versus groups I and II (*p* < 0.005; coefficient of determination, *R* ^2^ = 11.6 %).

MAP values before, during and after surgery are shown in Table 1 for all three study groups. In patients with normal blood pressure at baseline, differences in MAP values were not statistically significant (*p* > 0.05). In patients with treated hypertension, blood pressure decreased (in both men and women) during surgery, although these values returned to pre-surgical levels after surgery. Notwithstanding these changes, the observed differences in MAP were not statistically significant (*p* > 0.05). In patients with untreated hypertension, blood pressure normalized (i.e. became non-hypertensive) during surgery in both men and women, but returned to pre-surgical levels after surgery; however, this variation in blood pressure was not significant (*p* > 0.05).

**Table 1:** Mean arterial pressure (MAP) in the pre-, peri- and postoperative periods (mm Hg)

Arterial pressure	Preoperative period								Perioperative period								Postoperative period							
	Systolic				Diastolic				Systolic				Diastolic				Systolic				Diastolic			
	MAP		SD		MAP		SD		MAP		SD		MAP		SD		MAP		SD		MAP		SD	
	F	M	F	M	F	M	F	M	F	M	F	M	F	M	F	M	F	M	F	M	F	M	F	M
Patients with normal blood pressure	107.4	115.2	20.2	14.6	67.0	80.4	9.5	8.5	120.9	121.4	15.3	14.4	71.8	73.6	11.2	9.9	127.7	125.5	15.7	13.6	74.3	81.0	12.0	9.5
Patients with treated hypertension	136.3	140.4	9.8	12.9	87.8	85.7	8.1	9.7	121.0	128.0	13.5	14.6	64.4	72.6	13.5	9.9	141.6	140.7	14.2	13.2	82.3	83.0	9.2	9.9
Patients with untreated hypertension	143.0	139.1	12.4	11.5	84.7	86.7	7.3	9.8	126.2	128.4	14.2	15.6	77.3	78.7	12.2	11.5	140.8	144.1	17.5	16.2	78.7	86.7	9.4	9.8

As Table 1 shows, MAP after surgery was higher than the baseline values before surgery (regardless of the type of anaesthesia); however, these differences were not significant (*p* > 0.05). The greatest differences in MAP values occurred in patients with treated and untreated hypertension (Table 2).

**Table 2:** Mean heart rate (HR) in the pre-, peri- and postoperative periods

Heart rate	Preoperative period				Perioperative period				Postoperative period			
	HR		SD		HR		SD		HR		SD	
	F	M	F	M	F	M	F	M	F	M	F	M
Patients with normal blood pressure	77.1	79.1	8.5	10.4	80.4	81.7	9.7	10.4	73.8	75.9	12.6	10.2
Patients with treated hypertension	81.9	84.2	13.4	12.9	81.7	83.5	11.8	8.6	76.4	77.1	14.2	11.8
Patients with untreated hypertension	83.5	85.8	10.4	12.2	84.1	84.2	12.6	16.2	78.4	80.2	10.6	9.6

## Discussion

All of the outcome variables evaluated in this study—surgical time, anaesthesia time, total blood loss and mean blood loss rate—were lower in the group treated with TIVA. These findings strongly suggest that TIVA is superior to conventional anaesthetic techniques for controlling perioperative bleeding and is a strong argument for the use of TIVA in endoscopic paranasal sinus surgery.

Surgical treatment of massive mucosal inflammation of the nose and paranasal sinuses with polyps is particularly challenging, even when endoscopic methods are used. A two-stage surgical procedure is recommended, especially in patients who have previously had polyps removed from the nasal meatus (due to the possibility of adhesions), or when topographical identification of the surgical field is impeded. Local bleeding, which is difficult to control due to anatomical and pathological characteristics, affects the visibility of the surgical field during FESS. Excessive bleeding—which can be evaluated by the quantity of blood suctioned from the surgical field—is often a signal to interrupt surgery [9]. Therefore, it is important that anaesthesiology teams pay close attention to the hemodynamic parameters of the cardiovascular system, in addition to the usual monitoring of appropriate ventilation and analgesic procedures.

According to the United States National Library of Medicine, controlled hypotension is defined as a pharmacologically induced reduction of the systolic blood pressure to 80–90 mm Hg, a reduction of MAP to 50–65 mmHg or a 30 % reduction in baseline MAP. Controlled hypotension can be induced by patient-controlled epidural anaesthesia and a number of different hypotensive drugs. Of the various hypotensive medications available, vasoactive drugs (nitroglycerin, beta blockers, calcium channel antagonists) and clonidine and ACE inhibitors have proven to be the most effective. However, controlled hypotension is best achieved with precisely dosed, modern anaesthetic drugs, which have an immediate impact on hemodynamic parameters [10–12]. The combinations of remifentanil and propofol or remifentanil and inhaled anaesthetics (isoflurane, desflurane or sevoflurane) seem to be ideal due to their good safety profile and the ease with which the precise dose can be prepared and delivered. Moreover, these drugs do not accumulate in the body and have no impact on post-anaesthetic recovery. In addition, their use ensures that consciousness and psychomotor functions are recovered quickly upon termination of anaesthesia administration. Fentanyl, which is also commonly used, has an important drawback: it does not allow for a precise manipulation of haemodynamic parameters and can accumulate in the body depending on the dose applied [13–15].

A major limitation of the conventional administration of intravenous drugs is that even though the total drug dose administered is determined by the anaesthetist, its concentration in the brain depends on the volume and rate of distribution, the drug’s affinity for brain tissue and the speed of drug elimination from the body. Moreover, it is difficult to determine the appropriate drug infusion rate to ensure the appropriate level of sedation [16, 17].

Due to the disadvantages associated with the conventional intravenous techniques described above, TIVA and similar techniques have become popular in recent times and offer a viable alternative to inhalational anaesthesia. The emergence and feasibility of TIVA is due to advances in the pharmacokinetic and pharmacodynamic properties of newer drugs such as propofol. These properties make these drugs ideal for delivery via continuous infusion. Similarly, new computer technology has allowed for the development of sophisticated delivery systems (target-controlled infusion) that enable anaesthetists to easily control intravenous delivery.

Advances in the field of anaesthesiology have led to greater patient safety. Control and modification of cardiovascular system parameters ensure that arterial blood pressure and heart rate remain stable, thus facilitating good visibility of the surgical field, which in turn enables surgeons to work with greater confidence during surgery. In Poland, nearly all laryngological centres perform surgery under general anaesthesia, and use of this type of general anaesthesia has become so widespread that it can now be considered the standard in laryngological surgery.

Sieśkiewicz et al. [4] evaluated the relationship between mean arterial pressure and perioperative bleeding during FESS in patients with a low heart rate. They found that intraoperative bleeding is largely a function of MAP and HR: when the HR is maintained at 60 beats/min, there is no need, in many cases, to intensively reduce bleeding to achieve optimal surgical conditions. Surgery performed with drug-induced hypotension (arterial blood pressure of 50–60 mm Hg) does not always assure the desired effect of reducing perioperative bleeding caused by the diastole of peripheral vessels and automatic tachycardia, conditions that ultimately increase bleeding. Therefore, it is essential to prevent recurrent automatic tachycardia and to maintain heart rate at 60 beats/min [18, 19].

In that same study [4], Sieśkiewicz and colleagues used the Fromm and Boezzart scale [20] to evaluate perioperative bleeding and surgical field visibility. They found good results with an MAP between 65 and 78 mm Hg. However, generalized use of such a low blood pressure might be risky depending on the age of the patient; consequently, an individualized approach should be used. Theoretically, the decreased heart rate extends the diastolic duration and increases filling in the vessels, which ultimately results in increased cardiac output and bleeding in the operative field [18, 19].

A postoperative increase in arterial blood pressure, as occurred in our study, is usually associated with pain and for this reason analgesic treatment should be implemented in the intensive care unit (ICU). In our case, we did this by devising an algorithm to administer postoperative analgesics according to pain intensity levels. Using a visual analogue scale (VAS) ranging from 0 (no pain) to 10 scores (very severe pain), the intensity of the postoperative pain was divided into four categories, as follows: I—slight pain (VAS < 4); II—moderate pain (VAS 4–6, pain up to 3 days); III—severe pain (VAS 4–6, pain for over 3 days); and IV—very severe pain (VAS > 6). Depending on the patient’s medical history (hepatic failure, renal insufficiency, asthma, gastrointestinal disorders and blood clotting), we first administer non-opioid drugs, followed by opioids if the pain is long lasting.

## Conclusion

The use of technologically advanced dosing techniques (TCI) during entirely intravenous general anaesthesia in group III provides better control of hypotension, leading to less bleeding in the operative field, and a shorter operating time. Blood pressure in patients with hypertension (whether treated or not) should be pharmacologically normalized by an anaesthetist during the operation. However, this is often not possible and in such cases intensive bleeding will likely hamper the operation.

## References

[1] Ko MT, Chuang KC, Su CY (2008). Multiple analyses of factors related to intraoperative blood loss and the role of reverse Trendelenburg position in endoscopic sinus surgery. Laryngoscope.

[2] Baker AR, Baker AB (2010). Anaesthesia for endoscopic sinus surgery. Acta Anaesth Scand.

[3] Gorce BM, Ozkose Z, Tuncer B, Pampal K, Arslan D (2007). Hemodynamic effects, recovery profiles, and costs of remifentanyl-based anaesthesia with propofol or desfluran for septorhinoplasty. Saudi Med J.

[4] Sieśkiewicz A, Drozdowski A, Rogowski M (2010). The assessment of correlation between mean arterial pressure and intraoperative bleeding during endoscopic sinus surgery in patients with low heart rate. Otolaryngol Pol.

[5] Sivaci R, Yilmaz MD, Balci C, Erincler T, Uhul H (2004). Comparison of propofol and sevoflurane anaesthesia by means of blood loss during endoscopic sinus surgery. Saudi Med J.

[6] Wormald PJ, van Renen G, Perks J, Jones JA (2005). The effect of the total intravenous anaesthesia compared with inhalational anaesthesia on the surgical field during endoscopic sinus surgery. Am J Rhinol.

[7] Eberhart LH, Folz BJ, Wulf H, Geldner G (2003). Intravenous anaesthesia provides optimal surgical conditions during microscopic and endoscopic sinus surgery. Laryngoscope.

[8] Momota Y, Kaneda K, Arishiro K (2010). Changes in blood pressure during induction of anaesthesia and maxillofacial surgery by type and timing of discontinuation of antihypertensive drugs. Anesth Prog.

[9] Blackwell KE, Ross DA, Kapur P, Calcaterra TC (1993). Propofol for maintenance of general anaesthesia: a technique to limit blood loss during endoscopic sinus surgery. Am J Otolaryngol.

[10] Ahn HJ, Chung SK, Dhong HJ, Kim HY, Ahn JH, Lee SM, Hahm TS, Kim JK (2008). Comparison of surgical conditions during propofol or sevoflurane anaesthesia for endoscopic sinus surgery. Br J Anaesth.

[11] Nair S, Collins M, Hung P, Rees G, Close D, Wormald PJ (2004). The effect of β blocker premedication on the surgical field during endoscopic sinus surgery. Laryngoscope.

[12] Nekhendzy V, Lemmens HJ, Vaughan WC, Hepworth EJ, Chiu AG, Church CA, Brock-Utne JG (2007). The effect of deliberate hypercapnia and hypocapnia on intraoperative blood loss and quality of surgical field during functional endoscopic sinus surgery. Anesth Analg.

[13] Baker AR, Baker A (2010). Anaesthesia for endoscopic sinus surgery. Acta Anaesth Scand.

[14] Cafiero T, Cavallo LM, Frangiosa A, Burrelli R, Gargiulo G, Cappabianca P, De Divitiis E (2007). Clinical comparison of remifentanil–sevoflurane versus remifentanil–propofol for endoscopic endonasal transphenoidal surgery. Eur J Anaesth.

[15] Manola M, De Luca E, Moscillo L, Mastella A (2005). Using remifentanil and sufentanil in functional endoscopic sinus surgery to improve surgical conditions. ORL J Otorhinolaryngol Relat Spec.

[16] Crawley BK, Barkdull GC, Dent S, Bishop M, Davidson TM (2009). Relative hypotension and image guidance: tools for training in sinus surgery. Arch Otolaryngol Head Neck Surg.

[17] Danielson R, Gravningsbraten R, Olofsson J (2003). Anaesthesia in endoscopic sinus surgery. Eur Arch Otorhinolaryngol.

[18] Nair S, Collins M, Huang P (2004). The effect of beta-blocker premedication on the surgical field during endoscopic sinus surgery. Laryngoscope.

[19] Simpson P (1992). Perioperative blood loss and its reduction; the role of the anaesthetist. Brit J Anaesth.

[20] Boezzart AP, van der Merve J, Coetzee A (1995). Comparison of sodium nitroprusside and esmolol induced controlled hypotension for functional endoscopic sinus surgery. Can J Anaesth.

